# Medical Nutritional Therapy for Patients with Chronic Kidney Disease not on Dialysis: The Low Protein Diet as a Medication

**DOI:** 10.3390/jcm9113644

**Published:** 2020-11-12

**Authors:** Adamasco Cupisti, Maurizio Gallieni, Carla Maria Avesani, Claudia D’Alessandro, Juan Jesus Carrero, Giorgina Barbara Piccoli

**Affiliations:** 1 Department of Clinical and Experimental Medicine, University of Pisa, 56126 Pisa, Italy; dalessandroclaudia@gmail.com; 2 Nephrology and Dialysis Unit, ASST Fatebenefratelli Sacco, University of Milan, 20157 Milan, Italy; maurizio.gallieni@unimi.it; 3 Department of Biomedical and Clinical Sciences “Luigi Sacco”, University of Milan, 20157 Milan, Italy; 4 Division of Renal Medicine and Baxter Novum, Department of Clinical Science, Technology and Intervention, Karolinska Institutet, 14186 Stockholm, Sweden; carla.avesani@ki.se; 5 Department of Medical Epidemiology and Biostatistics, Karolinska Institutet, 17165 Stockholm, Sweden; juan.jesus.carrero@ki.se; 6 Department of Clinical and Biological Sciences, University of Torino, 10124 Torino, Italy; gbpiccoli@yahoo.it; 7 Nephrologie, Centre Hospitalier Le Mans, 72100 Le Mans, France

**Keywords:** low protein diet, chronic kidney disease, nutrition, dietary management

## Abstract

The 2020 Kidney Disease Outcome Quality Initiative (KDOQI) Clinical Practice Guideline for Nutrition in chronic kidney disease (CKD) recommends protein restriction to patients affected by CKD in stages 3 to 5 (not on dialysis), provided that they are metabolically stable, with the goal to delay kidney failure (graded as evidence level 1A) and improve quality of life (graded as evidence level 2C). Despite these strong statements, low protein diets (LPDs) are not prescribed by many nephrologists worldwide. In this review, we challenge the view of protein restriction as an “option” in the management of patients with CKD, and defend it as a core element of care. We argue that LPDs need to be tailored and patient-centered to ensure adherence, efficacy, and safety. Nephrologists, aligned with renal dietitians, may approach the implementation of LPDs similarly to a drug prescription, considering its indications, contra-indications, mechanism of action, dosages, unwanted side effects, and special warnings. Following this framework, we discuss herein the benefits and potential harms of LPDs as a cornerstone in CKD management.

## 1. Introduction

The type and quality of the diet that a patient follows is not trivial; it is a cornerstone in the management of many diseases, as it is the case of chronic kidney disease (CKD) [1]. The main goals of nutritional therapy in CKD are to avoid the accumulation of molecules that can reach toxic levels, because of impaired kidney elimination, to counterbalance the pro-catabolic milieu that characterizes advanced CKD stages, to reduce the generation of nitrogen compounds, and thus the retention of nitrogen waste products [2,3]. Dietary protein restriction can prevent and/or correct signs and symptoms of kidney failure, delay the time to kidney replacement therapy, and, perhaps counter-intuitively, prevent the development of protein–energy wasting (PEW) [4,5]. A low protein diet (LPD) may offer further metabolic benefits, including a better control of phosphate balance (important for the bone-mineral disease linked to CKD), provision of a higher dietary alkaline load (which can counteract metabolic acidosis), and potentially better control of other aspects or complications of progressive CKD, such as dyslipidemia, proteinuria, and inflammation [6].

The rationale, safety, and efficacy of LPDs delaying the start of dialysis are supported by various trials, leading to propose them with an evidence graded as 1A in the 2020 National Kidney Foundation-Kidney Disease Outcome Quality Initiative (NKF-KDOQI) Clinical Practical Nutrition Guideline [7]. However, LPD implementation is not widespread because of various fears and misconceptions. One concern is that LPD may induce PEW [8,9], but neither observational studies [10,11] nor randomized controlled trials [12] support such concern, within a well-planned and monitored low protein regimen. Instead, PEW is most likely part of the natural history of uremia rather than a direct complication of dietary restriction [13,14]. Conversely, careful nutritional assessment and wise and adapted dietary prescriptions, supplying enough energy to cover needs, may prevent PEW since LPDs can reduce intoxication and correct several metabolic abnormalities and symptoms.

Another commonly reported concern is that patients will not comply with dietary prescriptions [15]. While not all patients are suited to follow a LPD, most of them will adhere and comply with the treatment when given adequate information on its rationale and instructed about food choices. Implementation of shared decision-making, careful selection of the patients, availability of resources, and dedicated interdisciplinary teams (including nephrologists, dieticians, nurses, psychologists, and social workers) may facilitate adherence [16,17].

In CKD clinics, medical nutrition therapy (MNT) has been often considered as an ancillary lifestyle intervention whose role is somehow subordinated to pharmacological treatment. In this review, we would like to challenge this view and argue that MNT should be a cornerstone of the management of CKD as well as of other chronic diseases. The American Heart Association recently claimed diet quality to be the main predictor of death in the U.S. and that it should not be treated as anything less than a vital sign [18].

By definition, a medication is “something that treats, prevents, or alleviates the symptoms of disease” (https://www.thefreedictionary.com/medication); LPDs fulfill these criteria, with expected favorable effects, contraindications, and adverse reactions. We believe in the impact of MNT on the patient’s health and consider the implementation of LPD diets equal to the prescription of a medication.

Here, we discuss the importance of LPDs in CKD through the lens of a pharmacological approach, and summarize the mechanisms of LPDs’ action, indications, contra-indications, dosages, unwanted side effects, and special warnings. Acknowledging this concept is essential to ensure the success and safety of a dietary plan within a tailored and patient-centered MNT.

## 2. What Is a Low Protein Diet

A LPD is defined as a diet providing less than 0.8 g/kg b.w. (body weight)/day of protein [Food and Nutrition Board of the Institute of Medicine, National Academy of Science]. This threshold of intake is recommended by the U.S. Recommended Dietary Allowances (RDA) (https://www.ncbi.nlm.nih.gov/books/NBK234926/). RDAs are established from the minimum daily requirement covering the daily needs of 95% of the population; in the case of proteins, this is set as 0.46 g/kg b.w./day. This threshold is calculated by considering the intake of proteins of “high biological value”. The proteins at high biological value are those that provide all the essential amino acids, and traditionally this term has been considered as synonymous of animal proteins. From such minimum daily requirement, the RDA added 30% in acknowledgement of the consumption of proteins of high and low biological value within the diet, and another 30% to ensure safety. This is how the RDA of 0.77 g of protein/kg b.w./day was reached, and then approximated to 0.8 g/kgb.w./day.

The primary sources of animal proteins include beef, poultry, fish, eggs, milk and dairy products, and others. The list also contains processed food, like processed meats (sausages, ham, smoked turkey, bologna, and some types of cheese).

When prescribing a LPD, the intake of such foods tends to be discouraged, and thus LPDs are generally compatible with the concept of plant-based diets, as they result in a higher consumption of plant-derived foods, namely legumes, vegetables, cereals, and tubers [19].

Because of other healthy nutrients present in plants, this switch may result in additional benefits, beyond protein restriction. Conversely, reducing the intake of animal-derived foods and of ultraprocessed foods (especially when added with phosphate containing additives) [20], reduces unhealthy nutrients, such saturated fat, sodium, phosphate, and potassium.

LPDs must ensure adequate energy intake, which is estimated by the 2020 KDOQI guidelines [7] as 25–35 kcal per kg of ideal body weight per day. This figure, which is lower than the previous recommendations of 30–35 kcal/kg b.w./day [21], is the result of acknowledging both the older age of patients with CKD and the fact that the correction of anemia and acidosis is nowadays more effective than at the time in which previous thresholds were set, at least partly counterbalancing the hypercatabolic state of advanced CKD.

The way to calculate the energy and protein needs should be based on clinical judgment, as advised by the NKF-KDOQI Clinical Practical Nutrition Guideline [7]. If the patient’s body mass index is normal or overweight, the actual body weight is used to calculate energy and protein needs.

If the patient is obese or underweight, simply using a normative table of ideal body weight may not be enough. The clinical assessment includes evaluation of the body weight that the patient has maintained during adult life, the body weight that the patient considers adequate, and the body weight that minimizes the risk for developing complications from obesity or the risk for PEW. Therefore, normative body weight tables, respecting country, or ethnicity whenever possible, should not be the only parameter to calculate energy and protein needs.

## 3. Types and Dosages of Low Protein Diets

The requisite of the low-protein regimes is to ensure an adequate energy intake (25–35 kcal/kg b.w./day) [7]. Although many LPDs are possible in clinical practice, the following combinations (Table 1) are the most frequently reported in the medical literature, at least in western countries [22,23].

### 3.1. Conventional Low Protein Diet

The conventional LPD contains 0.6 g/kg b.w./day of proteins, of which at least 50% are of high biological value, such as egg, fish, and meat [24]. Besides ensuring that energy requirements are met, these diets should provide carbohydrates (contributing to 55–60% of the total energy intake), and lipids (contributing to 30–35% of the total energy intake). By limiting protein-rich foods (including dairy products and processed foods), this diet is also usually low in phosphate (600–800 mg/day), sodium (2–3 g/day), and calcium. By avoiding the intake of ultra-processed food (i.e., industrial ready-to-eat food, like ham, sausages, some types of cheese, smoked turkey, etc.) and increasing the intake of whole or minimally processed foods (fresh fruits and vegetables, cereals. And grains), this diet has the additive benefits described earlier.

Combining a low protein intake with a normal-to-high energy intake may be easier with the use of protein-free products that may represent a main energy source [25]. These products include pasta, noodles, bread, biscuits, flour, and precooked soups and desserts. This diet choice is particularly frequent in Italy, where these foods are reimbursed by the national health service [24])

### 3.2. Vegan Low Protein Diet

The vegan LPD provides 0.6 to 0.7 g/kg b.w./day. It is an option where protein-free products are not available or when the patient refuses to use them [24]. The crucial point is to cover the needs for essential amino acids in the presence of a protein intake lower than the RDA. To reach this goal, the diet is based upon combinations of cereals and legumes to complement the essential amino acids [26]. In fact, overall cereals and legumes are respectively poor in lysine and methionine; however, since cereals are rich in methionine whereas lysine is well represented in legumes, mixtures of cereals and legumes are complementary and nutritionally adequate.

### 3.3. Supplemented Plant-Based Diet

The supplemented plant-based LPD supplies 0.6 g/kg b.w./day of unselected mainly plant-derived proteins, supplemented with essential amino acids and/or ketoacids (1 tablet every 8–10 kg of body weight; the composition is available in the supplemental material). This dietary regimen includes only plant-derived foods (fruits, vegetables, sugars, starches such as pasta and bread, couscous, polenta, and legumes) supplemented with amino acids and ketoacids. The supplementation allows choosing any type of plant-derived food without the need to integrate different types of vegetable proteins (grains and legumes at each meal). The supplement intake depends on the body weight, type of diet, and nutritional status. The tablets should preferentially be taken during the meal. One to three unrestricted meals per week are usually allowed, to reduce diet tiredness and minimize the risk for nutritional deficits [27].

### 3.4. Very Low Protein Diet

The very low protein diet (VLPD) contains about 0.3 g/kg/day of proteins, supplemented with a mixture of essential amino acids and ketoacids (1 tablet for every 5 kg of body weight). This dietary regimen is usually achieved with a vegan diet. The target is usually limited to selected patients with stage 5 CKD and with high dietary compliance. It is generally implemented when conventional or vegan LPDs are not sufficient to maintain the metabolic homeostasis and the start of dialysis is not advisable, or not chosen by the patient [28,29,30].

## 4. Mechanism of Action of Low Protein Diets

The LPDs work, because they reduce the production of protein-derived waste products, the retention of protein-derived toxins and fixed acids, lower single nephron glomerular hyperfiltration and proteinuria, and reduce dietary phosphate load.

LPDs reduce the amount of protein-derived waste products and fixed acids, contributing to kidney protection and to the attenuation of metabolic abnormalities [1,2]. At each level of kidney function, a lower generation of protein–waste products lead to lower blood levels of uremic toxins, phosphate, and fixed acids.

In the kidney, a lower dietary protein load results in a reduction of single nephron hyperfiltration and glomerular hypertension, as well as trans-glomerular protein traffic [31].

Protein sources (i.e., plant or animal protein) may be relevant, but the evidence in this regard is scant. Early studies suggested that the favorable changes in glomerular hemodynamics may be more marked with plant-derived rather than animal-derived proteins [32].

**Figure 1:**
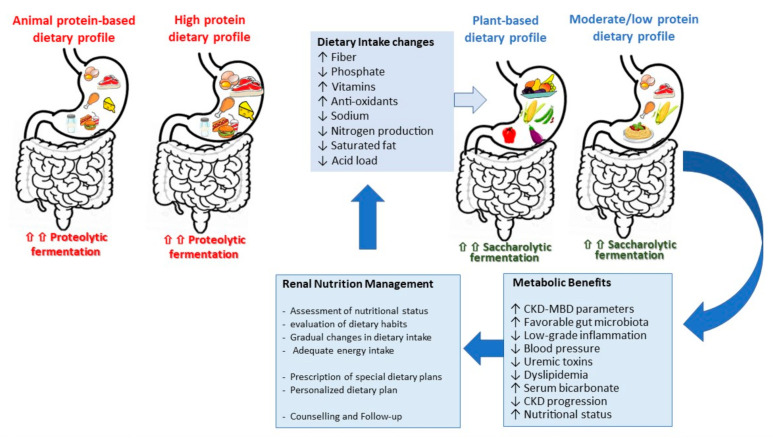
Renal nutritional management in chronic kidney disease (CKD) patients, on stages 3 to 5 not on dialysis: shifting from a high protein diet to a low protein and/or plant-based diet. CKD-MBD = Chronic kidney disease mineral and bone disorders.

The increased intake of complex carbohydrates and fibers contributes to establishing a favorable balance in the gut microbiota, favoring beneficial bacterial species with predominant saccharolytic metabolism (Figure 1) [33,34]. These micro-organisms ferment complex carbohydrates and fibers, producing short-chain fatty acids, such as acetate, butyrate, and propionate that serve as fuels for the intestinal epithelium and may have positive immune-modulatory effects. The reduction of urea limits its diffusion into the intestinal lumen, counteracting the vicious cycle of changes in intestinal microbiota, increase in intestinal permeability, and passage of bacterial toxins in the circulation [35].

## 5. Indications of Low Protein Diets

The main indications of LPDs are to delay the start of renal replacement therapy, the prevention and treatment of metabolic and electrolyte abnormalities, the prevention of protein–energy wasting, and the improvement of patient quality of life by reducing uremic symptoms, an additive contribution to the management of proteinuria, hypertension, and dyslipidemia (Figure 1).

Delay of the start of renal replacement therapy is a primary objective of LPDs. Besides the obvious advantages in terms of quality of life and cost of treatment, delaying dialysis may actually increase life expectancy, as recently underlined by the KDIGO guidelines [36]. LPDs may delay the start of dialysis in two ways: in the long run, the reduction of single-nephron hyperfiltration and proteinuria may slow the rate of decline of the residual kidney function; in the short run, LPDs improve the metabolic balance, reducing uremic symptoms and maintaining the nutritional balance, thus allowing to postpone dialysis, even if at stable glomerular filtration rate (GFR) [2,37]. Table 2 summarizes the main randomized controlled trials (RCT) investigating the role of protein restricted regimens on retarding the deterioration of renal function in CKD patients. In order to build this table, RCTs including at least 200 patients and with follow-up of at least 6 months were selected. Although the main findings of these studies separately do not support the role of low-protein regimens on retarding the progression of the disease, a recent meta-analysis including 16 studies showed that when pooling these studies together with others with smaller sample sizes together, the risk of progression to End Stage Kidney Disease(ESKD) was lower in patients receiving low protein regimens [38].

LPDs contribute to the prevention and control of various metabolic and electrolyte abnormalities, signs, and symptoms of CKD (Figure 1). These benefits are not necessarily mediated by the effect of protein restriction, but by the reduction of electrolytes and acids that usually accompany animal protein and ultraprocessed foods. By consuming more plants, LPD provide a higher intake of fibers, bioactive components, vitamins, antioxidants, and a more alkaline diet. This may have implications in blood pressure control and management of metabolic disturbances such as hyperphosphatemia, hyperkalemia, metabolic acidosis, and dyslipidemia. A secondary analysis of the Modification of Diet in Renal Disease (MDRD) study showed that a reduction of 0.2 g/kg b.w./day of protein per year was associated with lower serum concentrations of urea and phosphate, and higher levels of bicarbonate in patients with CKD stages 3–5, randomized to LPD or very low protein diet (VLPD) + keto analogues [39]. Cianciaruso et al. showed that patients following a LPD (0.6 g/kg b.w./day of protein) had lower serum urea nitrogen and needed lower bicarbonate supplements, phosphate binders, allopurinol, and diuretics [40]. The effect of LPDs in maintaining bicarbonate levels and lowering serum urea is consistent, and confirmed by a recent meta-analysis of 16 randomized controlled trials [38].

Furthermore, in patients with advanced CKD not on dialysis, a higher fiber to protein ratio was associated with lower concentration of uremic toxins derived from the gut microbiota (p-cresyl sulfate and indoxyl-sulfate), suggesting the selection of a healthier microbiota [45,46].

Almost paradoxically, LPDs can contribute to the prevention of PEW. The pathogenesis of PEW in CKD is multifactorial [8,9], but usually combines hypercatabolism and insufficient food intake. Loss of appetite is linked to the accumulation of nitrogen waste products, altered concentration of appetite regulators (such as ghrelin or cholecystokinine), dysbiosis, and local alterations such as periodontitis, and gastritis. Inadequate intake can also result from excessive restrictions that may leave the patient with few options to allow a healthy diet. Hypercatabolism is driven by a combination between chronic pro-inflammatory state, insulin resistance, metabolic acidosis and secondary hyperparathyroidism. These conditions result into a loss of muscle mass and fat stores [47].

By reducing production and retention of uremic toxins, low protein diet have the potential of reducing waste-products contributing to anorexia; furthermore, plant-derived foods contribute to reducing the net acid production, favoring the acid-base control [48].

Low protein intake can reduce proteinuria both of nephrotic and non-nephrotic range, and this effect is additive to the inhibition of the renin–angiotensin–aldosterone system (RAASi) [49,50]. The additive effect is explained by the different site of action: Reducing protein intake, especially of animal origin, reduces the afferent arteriole dilatation, whereas the RAASi lowers the resistance of the efferent arteriole. Both modifications reduce intraglomerular pressure and protein leakage. These favorable changes are also reported in iso-nitrogen load of plant-derived proteins.

The reduced sodium intake often associated with LPDs contributes to lowering arterial blood pressure [51]. These effects are additive to drug effects and reinforce the nephro-protection.

## 6. Contraindications to LPDs

**Figure 2:**
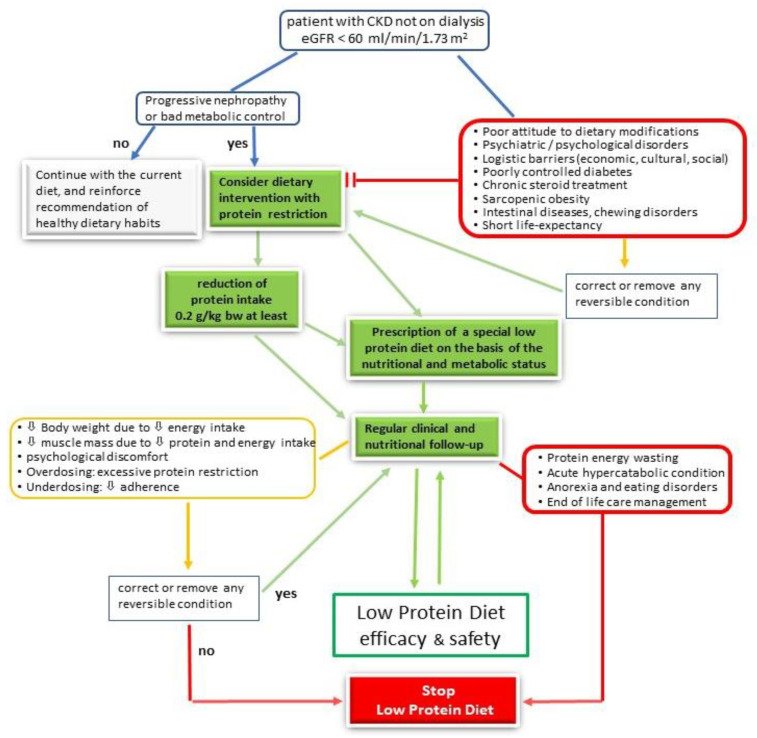
Low protein diet as medication: a flow chart for prescription.

Some contraindications to low protein diets exist, and they may be considered as absolute or relative (Table 3, Figure 2). As absolute contraindications may be assumed as acute hypercatabolic conditions, protein energy wasting, anorexia nervosa and eating disorders, and end of life care.

Acute severe diseases increase protein requirements, and thus represent an absolute contraindication to a LPD. High circulating levels of cytokines and pro-inflammatory molecules increase protein catabolism to face the acute event. In these settings, low protein intake can blunt the stress response and enhance the loss of lean body mass.

PEW, characterized by signs of muscle wasting, inability to spontaneously reach daily energy/protein targets, malnutrition as assessed by subjective global assessment or other validated scores, requires interventions aimed at restoring caloric intake, without protein restriction.

Eating disorders are usually considered absolute contraindications to low protein diets, to avoid interference with the diet management aimed at controlling the underlying disease. Information is scant as these patients are routinely excluded from trials and observational studies on LPDs [52].

In end of life care, starting a low protein diet is considered futile, unless aimed at symptom control (see specific paragraph below).

As relative contraindications, we consider most of the barriers (economic, cultural, lack of support, and so on) to LPD implementation, psychiatric disorders, poorly controlled diabetes, chronic steroid treatment, chronic gastro-intestinal diseases, including chewing disorders and short life-expectancy.

Relative contraindications to initiate or continue a LPD include both specific diseases and conditions that impair adherence to a diet, but that may be overcome, corrected, or removed. This latter category includes socio-economic issues, depression, psychological distress, chewing disorders, lack of motivation or of family and social support. To overcome these barriers, dietary prescriptions should be carefully monitored and, when needed, mitigated in particular during periods of clinical or physiological distress.

In patients with psychiatric disorders, including depression, a restrictive diet may increase distress; in these cases, attention should also be paid to the development of compulsive obsessive behavior, with the risk of over-restrictive attitudes, ultimately inducing malnutrition.

With regard to specific diseases or treatment frequently associated with CKD, chronic steroid treatment, and poorly controlled diabetes are often cited. High-dose, or long-term corticosteroid therapy increases protein catabolism, and induce sarcopenia; while full-blown steroid induced Cushing syndrome is becoming rare, indolent pictures are frequent and represent one of the reasons why LPDs are seldom prescribed in kidney transplant patients. Poor glycemic control in diabetic patients increases protein requirements and prevents adaptation of nitrogen metabolism to low protein intake [53,54]. The same is the case for severe metabolic acidosis.

Sarcopenic obesity, is not uncommon in advanced CKD [55], and there are no clear indications on how to combine energy reduction (given that obesity is a risk factor for CKD progression with protein restriction [56]. Chronic gastro-intestinal diseases and chewing disorders require particular attention, with the need to carefully advice on food choices.

Finally, short-life expectancy may also be considered a relative contraindication to starting or maintaining protein restriction, since changing dietary habits or restricting diet could be deranging and futile.

## 7. Unwanted Side Effects of LPD

Weight loss due to inadequate energy intake, Loss of muscle mass due to inadequate protein and energy intake. Depression, relational problems, psychological discomfort, overdosing(excessive protein restriction) or underdosing (ineffective protein restriction) (Table 3).

Unwanted weight loss is one of the most important concerns in patients on a LPD. Reduction of body weight, when not attributed to reduced fluid overload, means inadequate energy intake that is invariably associated with increased nitrogen need and prevents achieving the neutral or positive nitrogen balance that should characterize a well conducted LPD [53,57]. Loss of muscle mass may occur both when protein intake is adequate, but energy intake is insufficient and when the intake of protein and amino acid is inadequate. Importantly, a sedentary lifestyle is strictly associated with sarcopenia, and may enhance the loss of lean body mass. Long term adherence may be reduced by “diet tiredness”, associated with overzealous restrictions, use of poor tasty products, monotony, loss of appetite, potentially resulting in weight loss or in loss of focus, and reduced compliance.

Psychological discomfort and depression may occur as a result of the many hurdles CKD patients face in the course of their disease. Older patients may suffer from lack of appetite, sometimes aggravated by social isolation; chewing problems may reduce food choices and food variety may be limited by a small budget. Younger patients who work full time may need to regularly take eat meals outside home, where food choices are not always easy, or feel socially limited in their leisure time. In this regard, understanding the importance of LPD and knowledge of food choices, level of health literacy and coping capacity are important determinants of adherence [58]. Individualized dietary programs, intensive educational interventions, and regular counseling are needed to promote adherence to LPD.

Over-dosing may occur. Excessive protein restriction (possibly leading to insufficient energy intake) carries a high risk of PEW and of various vitamin and micronutrient deficits.

Under-dosing may also occur, linked to lack of dietary adherence, which obviously limits or impairs the efficacy of LPDs. A stepwise approach to protein restriction may help reaching targets and gaining confidence in the dietary modification (see implementation strategies).

In the presence of unwanted effects, the decision to continue the LPD depends on the severity and potential reversibility of the side effect. Shared decision-making and careful multidisciplinary follow-up are very important in these contexts.

## 8. Dietary Supplements

In some patients, nutritional supplements may be required to ensure the nutritional safety of a LPD and are mandatory in the case of a VLPD [59]. They include a mixture of essential amino acids and ketoanalogues, calcium, vitamins and iron preparations.

The most widely used supplements are a mixture of calcium salts of essential amino acids and keto acids, currently available world-wide, with small differences in composition [59,60]. The total nitrogen content per tablet is 36 mg, and the calcium content is 50 mg (1.25 mmol). In Italy the product of choice is identical, except for the absence of tryptophan [61].

Regular monitoring of vitamins, calcium and iron is required to target further supplementations.

By reducing the intake of phosphate-rich foods, calcium intake is usually decreased, and supplementation with 500–1000 mg of calcium may be needed.

Low iron stores may be due to the lower bioavailability of iron from plant-derived foods, and oral supplementation may also be needed.

Vitamin B_12_ supplementation is usually necessary for patients on a vegan diet for a long period, while it is rarely necessary when a vegan diet is alternated to conventional LPDs that provide a daily amount of meat or fish.

## 9. Special Warnings

### 9.1. Nephrotic Syndrome

Up to the 1980s, nephrotic syndrome was considered a contraindication for a LPD. Afterwards, experimental and human studies showed that reducing dietary protein load decreased urinary protein excretion [48], and this effect was synergic with RAASi [50]. The anti-proteinuric effect of LPDs was associated with an amelioration of albumin homeostasis and an increase in plasma albumin levels [62,63].

The additive effect of LPD and RAASi is explained by the combination of afferent arteriole vasoconstriction, induced by LPDs and post-glomerular vasodilatation, induced by angiotensin-converting-enzyme inhibitors [49,50]. The effect may be more marked in plant-based diets [64,65].

### 9.2. Diabetes

Historically, LPDs were contraindicated in diabetic patients with diabetes, and, on the contrary protein intake was increased to reduce carbohydrate load; indeed, early studies suggested a risk of malnutrition when implementing a LPD in these patients [66,67,68,69]. However, data in the literature are conflicting and this contraindication may no more be true. At present, worldwide most of the CKD diabetic patients are affected by type 2 diabetes and, at least in Europe, intensely proteinuric nephropathies leave the place to vascular nephropathies [70].

A recent consensus [68] and the 2020 update of the KDOQI clinical practice guidelines for nutrition in CKD suggest caution in reducing protein intake in patients with diabetes, setting a higher target of protein restriction (0.8 g/kg b.w./day) [7]. These indications are also shared by the International Society of Renal Nutrition and Metabolism [71]. Of note, this target is set to ideal body weight, which may correspond to consistently lower figures when referred to real body weight in type 2 diabetes patients [7]. It is also worth mentioning that low-carbohydrate diets with high protein content are increasingly proposed for weight reduction. A randomized trial has been advocated to assess whether low-carbohydrate diets are safe in diabetic patients with CKD [72].

### 9.3. Obesity

Obesity is an independent risk factor for CKD and cardiovascular diseases. It contributes to physical limitations and decline of functional status and is associated with poor quality of life and higher morbidity and mortality in the pre-dialysis phase.

Although evidence exists about the survival advantage of obesity in dialysis patients [73], reaching a normal or near-normal body mass index is often required for being wait-listed for kidney transplantation. Energy restriction increases protein requirements, so that a low protein diet may not be able to maintain an adequate nitrogen balance, thus increasing the risk of PEW.

Thus, energy restriction is potentially conflicting with a low-protein diet and, as a consequence, the two approaches are rarely combined. Priorities should be set on a case by case basis [24]. When a low-protein diet is indicated in an overweight or obese patient, protein restriction should probably be prescribed with an energy intake covering at least the daily requirements per ideal body weight [5].

### 9.4. Very Old Age

The NKF/KDOQI guideline in nutrition and CKD makes no specific recommendation for the elderly but suggests to carefully monitor energy intake in this group of patients in which food intake is often reduced, to a variety of reasons. Protein anabolism is reduced in older individuals and geriatric societies usually advice increasing protein intake of 1.0 to 1.2 g/kg b.w./day in elderly individuals, thus particular attention should be placed in protein restriction in elderly CKD patients. [74]. Furthermore, elderly patients are usually considered resistant to change their dietary habits.

However, due to the high mortality and morbidity of elderly patients on dialysis, delaying or even avoiding it dialysis of particular importance.

Brunori et al. in one of the few randomized controlled trials available in elderly patients, showed that a supplemented VLPD was able to delay by about one year the need of dialysis in elderly patients with no consequence on morbidity and mortality [28,75]. While very old patients (usually defined as over 80 years of age) are seldom included in RCTs, good results, at least in terms of safety, are reported in observational cohort studies [76]. A careful evaluation of dietary habits, functional and cognitive status, and regular monitoring of the nutritional status may allow extending the indications to this wide subset of the CKD population, selecting patients who can benefit from LPDs and adapting dietary recommendations to their needs [76,77].

### 9.5. Pregnancy

Pregnancy in CKD is a condition in which the advantages of the diet for the mother have to be carefully balanced with the theoretical risk for intrauterine growth restriction [78,79,80,81].

While most of the experiences come from a single referral center, available data suggest that moderately protein restricted, plant-based diets, whenever possible supplemented with amino acids and their keto-analogues, may allow stabilization of kidney function in pregnancy [78,81]. These diets are associated with an almost paradoxical improvement in intrauterine growth, and a significant reduction of a combined outcome encompassing early preterm delivery (<28 gestational weeks) and birth weight < 10 percentile per gestational age [81].

Protein restriction reported in these experiences is mild, targeting 0.6 g /kg b.w./day in the first half of pregnancy and 0.8 in the second half, accounting for the usual increase in energy and protein requirements during the last phases of gestation. The supplementation is usually increased from 1 tablet every 10 kg in the first 20 weeks to 1 tablet every 5–8 kg in the second half of the pregnancy. The same approach, aimed at blunting pregnancy-related hyperfiltration and the consequent increase in proteinuria, has been employed in patients with relevant proteinuria but preserved GFR at start of pregnancy [81,82].

Strict surveillance, and multidisciplinary care is needed to combine the multiple requirements of pregnancy with the prescribed protein intake.

### 9.6. Neuromuscular Disorders

Patients with spinal cord injury are at risk of developing metabolic diseases, including cardiovascular diseases, dyslipidemia, diabetes, and CKD. The latter is not only linked to the higher cardiovascular risk but often results from chronic urinary infections, bladder dysfunction, and nephrolithiasis. Patients with spinal cord injuries tend to increase fat body mass, reducing lean body mass, due to the lack of physical activity. In the absence of evidence regarding the efficacy of LPDs in this particular population, an individualized energy restriction with a normalized (0.8 g/kg b.w./day) protein intake may represent a feasible dietary option [83,84,85].

### 9.7. Short Life Expectancy

The concept of short life expectancy is complex and the term ambiguous, since it has been used for indicating periods of days-months (often less than 3 months), to longer periods, in particular when the term short is used with respect to the overall population.

Life expectancy of less than 3 months is considered a relative contraindication to the start of a classic nutritional approach, mainly based upon protein restriction, in patients with CKD stage 3–4, since death is expected before CKD progression, and changing dietary habits could be deranging and is expected to be futile.

When life expectancy is severely reduced but expected to be between 3 to 12 months, nutritional management may, on the contrary, have a fundamental role, in particular in cases with rapidly progressive CKD. In fragile, stage 4 and 5 CKD patients, the need of dialysis can be avoided, considering not only the fact that dialysis usually impairs quality of life more than a low-protein diet but also the excess mortality at dialysis start. Similar considerations may apply to the “palliative” care of patients with advanced CKD.

## 10. Implementation and Monitoring

A common strategy for reaching the target energy and protein intake has not been agreed upon, and each team should work according to socio-cultural habits and personal/center experience [86]. The following points are however commonly highlighted: assessment, stepwise approach, follow up, and personalization (Figure 2).

Assessment of the baseline dietary habits is the first very important step, as well as assessment of body composition and functional status. At the beginning of treatment, especially when the current protein intake is high, it is reasonable to progressively reduce the protein intake by subsequent steps, indicatively of 0.2 g/kg b.w./day trying to normalize protein intake as a first goal (0.8 g/kg b.w./day) [39,76].

The target value can then be gradually reached by progressive adaptation of protein intake, based on the metabolic and clinical homeostasis and with a shared decision-making process involving the patient and the caregivers. Variety of the food choices may support adherence in the long-term. Different strategies have been suggested, including unrestricted meals, or alternating vegan diet and conventional low-protein diet [8,27,87,88,89].

Nutritional monitoring of patients on low protein diets aims to verify the adherence to dietary prescription and to early detect PEW, is any. The assessment of adherence is essentially a control of the stability of CKD: in this regard, we suggest monitoring of serum urea, phosphorus, parathormone (PTH), hemoglobin, bicarbonate, proteinuria and residual renal function. Expected effects of LPDs are the initial reduction and subsequent stabilization of serum urea, phosphate, PTH, stabilization of eGFR, and increase or stabilization of serum bicarbonate and albumin. Evaluation of 24-h urine excretion of sodium, phosphate, and urea allows estimating the respective dietary intakes.

As indicated also in the recent KDOQI guidelines, body composition should be controlled to timely identify PEW: body weight, subjective global assessment, malnutrition-inflammation score, serum albumin, serum cholesterol, and body composition analysis are the main assessments. Monitoring of diet diary and quality of life is also recommended [3]. Performance measures such as gait-speed or handgrip strength, are increasingly recommended.

Information and education are mainstays to facilitate adherence [15]. The form of the educational sessions is culture-sensitive and may include interactive presentations engaging the professional figures involved in the clinical management of CKD, whose aim is direct patient involvement in diagnostic and therapeutic decision-making processes [90,91,92].

## 11. Conclusions

MNT should not be considered only a lifestyle option in CKD, but instead a core element in its management. LPDs are effective therapies to delay the start of dialysis and have additional benefits beyond protein restriction by promoting better dietary quality. The degree of evidence recently identified for this treatment (1A) is similar to that of other cornerstone therapies like RAASi (graded 1B by KDIGO) [7], and as such a LPD should be prescribed. As with all medications, LPDs have indications and contra-indications, and require considering different approaches and evaluating the risk of unwanted side effects (Table 3, Figure 2).

Successful implementation requires motivation and close interaction between patients and members of an interdisciplinary team including physicians, nurses, dietitians, and social workers. In our experience, this kind of implementation of LPD can give more chances of efficacy and safety.

## Figures and Tables

**Table 1:** Main types and dosages of low protein regimens.

Type of Diet	Protein Restriction (g/kg of body weight per day)	Main Features
Conventional low protein diet	0.55–0.60 g/kg b.w./day; mixed protein	This regimen is a very popular choice in Italy, where protein-free food is provided free of charge to CKD patients. Protein-free pasta, bread, and other carbohydrates substitute the usual bread, pasta, or rice.
Vegan low protein diet	0.6–0.8 g/kg b.w./day; vegetable protein	Protein intake in vegan diets is on the average 0.7–0.9 g/kg/day; due to the different bioavailability, a 0.7 diet roughly corresponds to a 0.6 mixed protein diet.
Vegan supplemented (moderate restriction) Diet	0.6–0.7 g/kg b.w./day; vegetable protein, supplemented with essential amino- and keto-acids	A simplified prescription is based upon food to be avoided (animal origin) and preferred (vegetable origin) food. Animal-derived food is allowed only in unrestricted meals. Supplementation is tailored upon nutritional status and clinical situation (1 pill every 8 to 10 kg of ideal body weight)
Very low-protein supplemented vegan diet	0.28–0.43 g/kg b.w./day; vegetable protein only, supplemented, with or without protein-free food	This diet may be upon food to be avoided (animal origin) and preferred (vegetable origin) food. Animal-derived food is negotiated with the patient in few “free meals” (usually no more than 1 per week). Supplementation of ketoacids is higher (1 pill every 5 kg of ideal body weight). Carbohydrates are mainly from protein-free products in countries where this is available, as in Italy.
Tailored solutions	Usually 0.6–0.8 g/kg b.w./day, vegetable or mixed	These solutions employ different combinations of protein-free food, vegan diets. and supplementation to encounter patient’s taste and metabolic needs.

CKD = Chronic Kidney Disease.

**Table 2:** Summary of the randomized controlled clinical trials, including more than 200 patients with follow-up > 6 months, investigating the role of protein restricted diets in patients with chronic kidney disease of stages 3 to 5 (not on dialysis).

Author, Year	Aim and Outcome	Sample Characteristics	Main Findings	Comments
Rosman et al., 1984 [41]	Role of protein restriction in retarding CKD progression rate of decline of renal function.	228 pts (15 to 73 years old) GFR 30 to 60 mL/min/1.73 m^2^ LPD 0.6 g/kg/day vs. UPD GFR ≤ 30 mL/min/1.73 m^2^ LPD: 0.6 g/kg/day vs. UPD. 2-year follow-up.	The LPD groups showed lower decrease in serum Creatinine over time, whereas the UPD groups showed no changes. The LPD had a lower serum urea during the study when compared to the UPD group.	No changes in body weight and serum albumin were observed during the study in the LPD and UPD groups.
Locatelli et al., 1991 [42]	Role of protein restriction in retarding CKD progression. Renal survival (need for dialysis or doubling serum Creatinine).	456 pts (18–65 years old) GFR < 60 mL/min/1.73 m^2^ LPD: 0.6 g/kg/day vs. UPD: 1.0 g/kg/day. 2-year follow-up.	Renal survival was a lithe bit higher in the LPD group vs. UPD (*p* = 0.06). No differences in GFR were observed between the LPD and UPD groups.	Low adherence to the prescribed protein intake in the LPD group. No changes in body weight were observed during the study in the LPD and UPD groups.
Klahr et al., 1994 MDRD, Study 1 [43]	Role of protein restriction and blood pressure control in retarding CKD progression GFR rate of decline.	585 patients (18–70 years old) GFR 55 to 25 mL/min/1.73 m^2^. BP control level: Usual vs. Low LPD: 0.6 g/kg/day vs. UPD: 1.3 g/kg/day. 2.2-year follow-up	No differences in the rate of GFR decline between protein intake groups and blood pressure groups were observed.	Low adherence to the protein prescribed in LPD and UPD groups. Nutritional status was maintained thru the study in the LPD, UPD and VLPD groups.
Klahr et al., 1994 MDRD, Study 2 [43]	Role of protein restriction and blood pressure control on CKD progression GFR decline.	Study 2: 255 patients (18–70 years old) GFR 24 to 13 mL/min/1.73 m^2^. BP control level: usual vs. low LPD: 0.6 g/kg/day vs. VLPD: 0.3 g/kg/day plus supplementation of EAA and KA. 2.2-year follow-up	No differences in the average rate of GFR decline between protein and blood pressure groups were observed.	Although not significant, a trend to slower GFR decline was observed in the VLPD vs. the LPD group (*p* = 0.07).
Cianciaruso et al., 2008 [40]	Role of low protein diet on metabolism Modification in serum urea.	392 patients (Age > 18 years) GRF ≤ 30 mL/min/1.73 m^2^ LPD: 0.6 g/kg/day vs. UPD: 0.8 g/kg/day. 6 to 18 months follow-up.	No difference in serum urea between LPD and UPD group, but serum urea was lower in patients adherent to LPD. The need for medications was lower in the LPD vs UPD group.	Nutritional status was maintained in the LPD and UPD group.
Garneata et al., 2016 [44]	Role of severe protein restriction in retarding CKD progression. Dialysis start or 50% reduction of eGFR	207 pts median age 54 years eGFR *<* 30 mL/min/1.73 m^2^ and proteinuria *<* 1 g/day. LPD (0.6 g/kg/day) vs. VLPD (0.3 g/kg/day) plus supplementation of EAA and KA. 15-month follow-up	VLPD plus EAA and KA was more effective than LDP in delaying the start of dialysis or avoiding 50% loss of GFR, especially *n* patients with GFR < 20 mL/min.	Compliance to diet was good, with no changes in nutritional parameters. The randomization occurred in patients previously selected for attitude to dietary restriction during the run-in period.

LPD: Low protein diet; UPD: Usual protein diet; VLPD: Very low protein diet; GFR: Glomerular filtration rate; eGFR: Estimated glomerular filtration rate; BP: Blood pressure; CKD: chronic kidney disease; EAA: essential amino acids, KA: ketoacids.

**Table 3:** Low protein diets as a medication.

A. Types and Dosages of Low Protein Diet
- Conventional LPD, supplying 0.55 to 0.60 g/kg b.w./day of mixed proteins, at least 50% of high biological value. - Vegan LPD, supplying 0.6–0.7 g/kg b.w./day of plant origin proteins using a special combination of cereal and legumes, and/or soy. - Very low protein diet to patients with CKD and without diabetes: 0.28 to 0.43 g/kg b.w./day with additional keto acid/amino acids to meet protein requirement of 0.55 to 0.60 g/kg b.w./day, usually 1 tablet every 5 kg b.w. - Mostly vegetarian diets supplying 0.7–0.8 g/kg b.w.d of unselected proteins, supplemented or not with essential amino acids and ketoacids (usually 1 tablet every 10 kg b.w).
**B. Mechanisms of Action of Low Protein Diet**
- Reduced production of protein-derived waste products - Reduced retention of protein-derived toxins and fixed acids - Reduced phosphate load, with lesser stimulation of parathyroid hormone production - Reduced single-nephron glomerular hyperfiltration - Reduced urine protein excretion
**C. Indications to a Low-Protein Diet**
- Prevention and treatment of metabolic and electrolyte abnormalities, signs and symptoms of chronic renal insufficiency - Prevention of protein-energy wasting - Delay the start of renal replacement therapy - Management of proteinuria, hypertension, or progressing chronic kidney disease
**D. Contraindications to a Low-Protein Diet**
Absolute - Protein energy wasting - Hypercatabolic state (acute or chronic) - Anorexia and eating disorders - End of life care management
Relative - Poor attitude to dietary modifications - Psychiatric / psychological disorders - Logistic barriers (economic, cultural, lack of support) - Poorly controlled diabetes - Chronic steroid treatment - Intestinal diseases including chewing disorders - Short life-expectancy
**E. Unwanted Side Effects of LPD**
- Weight loss due to reduced energy intake - Loss of muscle mass due to inadequate protein and energy intake - Depression, relational problems, psychological discomfort
**F. Possible Supplementations during Low Protein Regimens**
- Calcium - Vitamins - Iron - Essential amino acids and ketoacids
**G. Special Conditions**
- Nephrotic syndrome - Diabetes - Obesity - Pregnancy - Very old, aged patient - Neuromuscular disorders - Short life expectancy
